# Fluorescence amplified fragment length polymorphism for subtyping of genotypes of *Acanthamoeba* isolated from patients with keratitis

**Published:** 2011-01

**Authors:** K. Prashanth, Gunisha Pasricha, Savitri Sharma

**Affiliations:** * *Department of Biotechnology, School of Life Sciences, Pondicherry University, Pondicherry, India*; ** *Centre for DNA Fingerprinting & Diagnostics, Hyderabad, India*; + *L V Prasad Eye Institute, Hyderabad Eye Research Foundation, Hyderabad, India*; # *Ocular Microbiology Service, L V Prasad Eye Institute, Bhubaneswar, India*

**Keywords:** *Acanthamoeba* keratitis, T4 genotype differentiation

## Abstract

**Background & objectives:**

*Acanthamoeba* keratitis (AK) is a painful and vision-threatening ocular infection. The differentiation of *Acanthamoeba* at the species and subspecies level is complicated. Nearly all the AK isolates have been shown to belong to T4 genotype when analysed by ribosomal RNA gene sequences and there is no universally acceptable method for differentiation of different subtypes of T4. The purpose of this study was to attempt further discrimination of T4 genotypes.

**Methods:**

In the present investigation, 15 *Acanthamoeba* isolates obtained from cornea of keratitis patients were subjected to fluorescence amplified fragment length polymorphism (FAFLP) genotyping to differentiate T4 subtypes.

**Results:**

FAFLP profiles showed five distinct clusters (I to V) within T4 clonal complex which clearly depicted genetic differences among the isolates of T4 sequence type of *Acanthamoeba*.

**Interpretation & conclusions:**

Our study demonstrated the usefulness of FAFLP for reliable differentiation of T4 clonal complex of *Acanthamoeba*.

*Acanthamoeba* comprises of free-living amoebae and certain species of this genus can cause amoebic keratitis, a painful and vision-threatening infection^1^,^2^. *Acanthamoeba* keratitis (AK) is characterized by a recalcitrant corneal ulceration. The differentiation of *Acanthamoeba* at the species level is complicated with at least 24 named species described within this genus^3^. Genotyping of *Acanthamoeba* isolates by partial or complete nuclear 18S ribosomal RNA gene (*Rns*) sequence analysis, random amplified polymorphic DNA (RAPD) and restriction fregment length polymorphism (RFLP) analysis of complete mitochondrial genome or mitochondrial 16S rRNA genes (*rns*) have been very useful^4^–^8^. So far, only five genotypes have been associated with keratitis, of which the majority have been T4 but T3, T2, T6, and T11 have each caused individual cases^1^,^9^,^10^. Though all the *Acanthamoeba* isolates from the cornea appear to be genotype T4, it is possible that different subtypes of T4 exist. Previous observations have noted that an inter-strain sequence difference within T4 is up to 0-4.3 per cent suggesting that subsets of T4 are involved in clinically important keratitis^5^.

Till now, there are no unambiguous means to reliably differentiate isolates within T4. Some investigators have attempted the sequencing of first internal transcribed spacer (ITS1) and *rns* of the mitochondria for further differentiation of T4^11^,^12^. However, these methods have their own limitations^11^. The amplified restriction fragment length polymorphism (AFLP) technique is a relatively new genotypic method that has not yet been widely used in parasitology^13^. The present study attempted to differentiate isolates within T4 sequence type of *Acanthamoeba* using FAFLP.

## Material and Methods

Fifteen *Acanthamoeba* isolates obtained from the corneal scrapings of patients diagnosed to have keratitis at LV Prasad Eye Institute (LVPEI), Hyderabad were used for DNA analysis. Prior ethical clearance was obtained from Institutional ethical committee for the study. The details of the isolates are given in the Table. One standard reference isolate (*Acanthamoeba castellani* ATCC 50370) was included as control. All isolates were grown axenically as monolayers in protease peptone-yeast-glucose medium^14^,^15^. The total DNA was extracted from the amoebic cultures according to the method described earlier^15^. Purity of DNA was checked and contamination by bacterial DNA was ruled out by performing a 16S ribosomal DNA PCR for eubacteria (for possible contamination by intracellular bacterial endosymbionts in *Acanthamoeba*). The isolates were subjected to 18S ribosomal DNA typing^15^and all were found to be T4 sequence genotypes. All isolates were subjected to FAFLP as described previously^16^,^17^. Using the enzyme combination of *EcoR*I- *Mse*I, we analyzed the genomic fragments that were distributed within the size range of 50 to 500 bp. Primer combinations used were *EcoR*I+0 and *Mse*I+C. FAFLP experiment and analysis (AFLP Microbial Fingerprinting kit; Applied Biosystems, California, USA) were done as per the manufacturer’s instructions. To investigate the stability and reproducibility of FAFLP, three different DNA preparations from the same isolate were subjected to the same FAFLP reaction conditions at different time intervals. Such exercise was carried out wherein the time interval was one month each between these preparations and PCR reactions.

In each case, DNA was double digested with the endonucleases and the resulting restriction fragments were ligated with the double-stranded adapters. The restriction ligation reaction was carried out simultaneously in a single step. Pre-selective PCR and selective PCR were carried out as described earlier^16^,^17^. Selective PCR products, along with formamide loading dye and the internal lane standard 6-carboxy-x-rhodamine (GS 500 Rox, PE Biosystems, California, USA) were loaded onto an ABI Prism 3100 DNA sequencer (Applied Biosystems, USA). Fragment separation was continued for 2.5 h through a performance-optimized polymer 4. Fragments were detected and compiled by the ABI Data Collection (Perkin-Elmer, Applied Biosystems) software. Electropherograms and fragment analysis were performed with the GeneScan software version 3.7. FAFLP electropherograms were analyzed using GeneScan 3.7 and Genotyper 3.7 soft wares (PE Biosystems, USA) to detect differentially amplified genomic fragments (amplicons). The presence or absence of amplicons within the categories was scored by a user-defined Genotyper macro. Allele scores (the presence or absence of amplicons) were converted into binary format. The percentage similarities/differences between FAFLP amplitypes were calculated using the Dice correlation coefficient. Genotyper binary data were converted in to a distance matrix and dendrograms were deduced using the UPGMA algorithm.

## Results & Discussion

A more precise differentiation of genotype would be beneficial for a better understanding of taxonomy of *Acanthamoeba* and this may also facilitate the correct identification. Whole cell DNA typing by FAFLP can fulfill these requirements. Molecular characterization of AK isolates using *Rns* DNA genotyping in our study showed all the 15 isolates to be T4 genotype and bear the typical T4 signatures that define the pathogenic forms. This was in concordance with the earlier studies^4^,^18^,^19^. The fact that AK isolates widely tested belonged to the T4 genotype suggests that these might also represent a limited subset of related genotypes^3^. Nevertheless, the Indian isolates within themselves showed considerable variation implying that T4 sequence type might be heterogeneous group comprising of multiple species^15^.

**Fig. 1:**
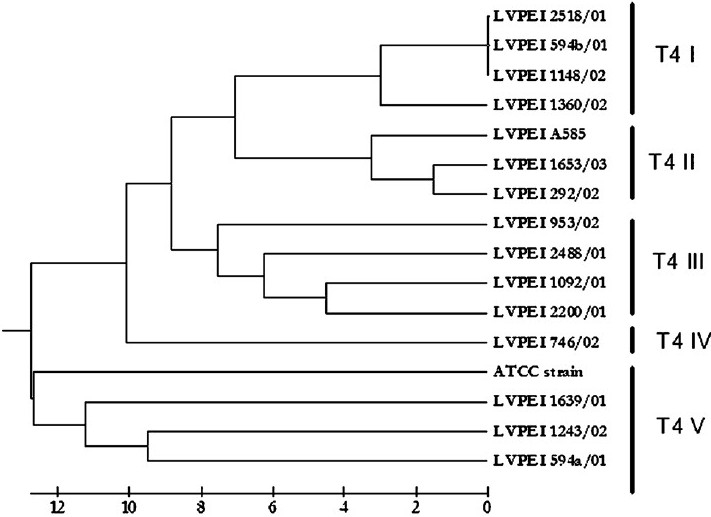
Dendrogram depicting the genetic distances among the strains of T4 sequence types constructed by UPGMA algorithm using binary data generated through the FAFLP profiles.

**Fig. 2:**
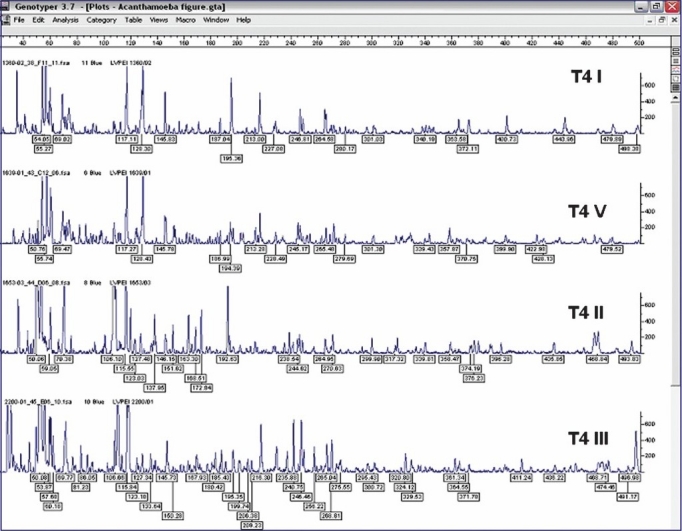
FAFLP electropherograms of representative strain subsets (T4 I - T4 V) of T4 sequence type depicting the range between 40-500 bp for *Mse*I+C selectivity tested, showing number of fragments and size of fragments as well as the peak heights.

In this study, we examined 15 isolates of T4 lineage along with one additional type strain of T4 lineage by FAFLP with a single primer combination. All isolates were from patients from Andhra Pradesh (between 2001 and 2003) except one from Maharashtra seen at LV Prasad Eye Institute. The predisposing factor in all patients was trauma and none was related to contact lens wear (Table). FAFLP generated a total of 76 to 114 differently sized fragments experimentally ranging in size from 50 to 500 bp for all the isolates. The dendrograms constructed by UPGMA showed five clusters which were designated using Roman numerals preceded by sequence type T4 (Fig. 1). The FAFLP genetic profiles of strains belonging to T4 showed substantial differences among themselves as witnessed by their electropherograms (Fig. 2).

**Table:** Details of the *Acanthamoeba* isolates obtained from corneal scrapings of keratitis patients

Strain ID	Date of isolation	Patient’s age/sex	Patient’s origin -Village/City/State
L-1092/2001	12-06-01	35/M	Moulangivillage, Nizamabad Andhra Pradesh
L-1639/2001	22-08-01	24/M	Sanathnagar, Hyderabad, Andhra Pradesh
L-2200/2001	16-11-01	30/M	Pillalamarri village, Nalgonda, Andhra Pradesh
L-2488/2001	27-12-01	19/M	Agamothkuru, Nalgonda, Andhra Pradesh
L2518/2002	01-01-02	63/M	Puljala village, Nagarkurnool, Mehabubnagar, Andhra Pradesh
L-292/2002	13-02-02	65/F	Hyderabad, Andhra Pradesh
L-746/2002	17-04-02	35/M	Yadagarpally, Nalgonda, Andhra Pradesh
L-953/2002	20-05-02	30/F	Nagarkurnool, Mahabubnagar Andhra Pradesh
L-1148/2002	19-06-02	55/M	Darmajigudem village, West Godavari, Andhra Pradesh
L-1243/2002	03-07-02	25/M	Rudravelu village, BB Nagar, Nalgonda,
L-1360/2002	16-07-02	35/F	Chinnakistapuram village, Medak Dist. Andhra Pradesh
L-594a/2002	26-03-02	30/M	Krishnalanka, Vijayawada, Andhra Pradesh
L-594b/2002	15-04-02	30/M	Krishnalanka, Vijayawada, Andhra Pradesh
L-1653/2003	08-08-03	30/M	Lathur, Maharasthra
L-A585/2003	14-09-03	36/F	Hyderabad, Andhra Pradesh

*Rns* sequence typing occasionally has complications caused by multiple alleles or introns present. The *rns* sequences are though shorter, consistent in length, have common occurrence of identical sequences among many strains which is presumably due to a peculiarity of the primer sequences used in this technique that may fail to differentiate minor variations among strains. FAFLP can be a newer option to genotype strains of *Acanthamoeba* particularly strains belonging to T4 sequence types. A single isolate belonged to T4-IV showed considerable genetic difference of 10 per cent when compared with other clones of T4 complex. FAFLP profiles of three isolates of T4 V in our study showed significant variation from all the sequence types of T4 isolates by having a genetic distance of more than 9 per cent and up to 11.5 per cent. Such genetic variations probably constitute isolates belonging to multiple species within the complex, which is yet to be investigated.

Only in two instances identical T4 subtype was isolated in the same geographical location. At Nalgonda, T4 subtype III appeared to be common and in Hyderabad city, T4 subtype II was isolated in two cases. However, in two patients from Mehabubnagar, T4 subtypes isolated were altogether different (Table). In each of these cases, source of the infections could not be traced. Interestingly, from a single patient two isolates (L-594a & L-594b) were isolated within a span of around 20 days shown to belonged to two different T4 subtypes (T4 V & T4 I) in the study. However, the reason is not clear.

*Acanthamoeba* isolates from keratitis belong to genotype T4 with a few exceptions^1^,^4^,^18^–^20^. In this study, FAFLP clusters were well associated with 18S rDNA sequence types^15^. However, dissimilarities between same sequence types could be recognized more easily by FAFLP than the *Rns* typing (Fig. 1). Therefore, FAFLP appears to be a potential candidate to distinguish between different sequence types (data not shown) or even within sequence type T4. PCR based AFLP typing is also cost-effective as it needs only fluorescent primers and adapters when compared to enormous chemical requirements of RNA sequence typing. FAFLP was also shown to be highly reliable and accurate in many studies^16^,^17^. However, chances of *Acanthamoeba* DNA contamination by DNA of intracellular bacteria, fungi and virus in *Acanthamoeba* cells might interfere with the FAFLP genome analysis, which should be ruled out before attempting the FAFLP for AK isolates.

The results of this pilot study show that FAFLP can be a useful tool for the genotyping of new isolates and the assessment of genetic relatedness of *Acanthamoeba* spp. FAFLP can also be used for identification of individual isolates that could trace the potential paths of AK infections. Also, the reliable genotyping of T4 clonal complex. FAFLP was successful in differentiating closely related strains of T4 genotype and it can also highlight the substantial degree of genetic diversity within this clonal complex. This study also provides evidence that T4 is the predominant genotype in India responsible for AK. Hence, FAFLP can be a time-saving alternative to expensive sequence analysis for identification of unknown *Acanthamoeba* isolates through comparison with known FAFLP profile database of different genotypes and their subsets.
